# On the pivotal role of PPARα in adaptation of the heart to hypoxia and why fat in the diet increases hypoxic injury

**DOI:** 10.1096/fj.201500094R

**Published:** 2016-04-21

**Authors:** Mark A. Cole, Amira H. Abd Jamil, Lisa C. Heather, Andrew J. Murray, Elizabeth R. Sutton, Mary Slingo, Liam Sebag-Montefiore, Suat Cheng Tan, Dunja Aksentijević, Ottilie S. Gildea, Daniel J. Stuckey, Kar Kheng Yeoh, Carolyn A. Carr, Rhys D. Evans, Ellen Aasum, Christopher J. Schofield, Peter J. Ratcliffe, Stefan Neubauer, Peter A. Robbins, Kieran Clarke

**Affiliations:** * Department of Physiology, Anatomy and Genetics, University of Oxford, Oxford, United Kingdom;; † Division of Cardiovascular Medicine, Radcliffe Department of Medicine, Wellcome Trust Centre for Human Genetics, University of Oxford, Oxford, United Kingdom;; ‡ Chemistry Research Laboratory, University of Oxford, Oxford, United Kingdom; and; § Nuffield Department of Clinical Medicine, University of Oxford, Oxford, United Kingdom

**Keywords:** cardiac contractile function, HIF, myocardial energy metabolism, ^31^P MRS, substrate metabolism

## Abstract

The role of peroxisome proliferator-activated receptor α (PPARα)-mediated metabolic remodeling in cardiac adaptation to hypoxia has yet to be defined. Here, mice were housed in hypoxia for 3 wk before *in vivo* contractile function was measured using cine MRI. In isolated, perfused hearts, energetics were measured using ^31^P magnetic resonance spectroscopy (MRS), and glycolysis and fatty acid oxidation were measured using [^3^H] labeling. Compared with a normoxic, chow-fed control mouse heart, hypoxia decreased PPARα expression, fatty acid oxidation, and mitochondrial uncoupling protein 3 (UCP3) levels, while increasing glycolysis, all of which served to maintain normal ATP concentrations ([ATP]) and thereby, ejection fractions. A high-fat diet increased cardiac PPARα expression, fatty acid oxidation, and UCP3 levels with decreased glycolysis. Hypoxia was unable to alter the high PPARα expression or reverse the metabolic changes caused by the high-fat diet, with the result that [ATP] and contractile function decreased significantly. The adaptive metabolic changes caused by hypoxia in control mouse hearts were found to have occurred already in PPARα-deficient (PPARα^−/−^) mouse hearts and sustained function in hypoxia despite an inability for further metabolic remodeling. We conclude that decreased cardiac PPARα expression is essential for adaptive metabolic remodeling in hypoxia, but is prevented by dietary fat.—Cole, M. A., Abd Jamil, A. H., Heather, L. C., Murray, A. J., Sutton, E. R., Slingo, M., Sebag-Montefiore, L., Tan, S. C., Aksentijević, D., Gildea, O. S., Stuckey, D. J., Yeoh, K. K., Carr, C. A., Evans, R. D., Aasum, E., Schofield, C. J., Ratcliffe, P. J., Neubauer, S., Robbins, P. A., Clarke, K. On the pivotal role of PPARα in adaptation of the heart to hypoxia and why fat in the diet increases hypoxic injury.

Each day, the human heart beats ∼100,000 times and pumps ∼10 tons of blood through the body, therefore requiring ∼6 kg of ATP (1). To produce such a large quantity of ATP, the heart relies on mitochondrial oxidative phosphorylation of a variety of metabolic fuels, including free fatty acids (FFAs) and glucose. In theory, FFAs require ∼13% more oxygen than glucose to generate the same amount of ATP, although hearts metabolizing FFAs may require far more oxygen, owing to increased mitochondrial uncoupling (2, 3). ATP is transported from the mitochondria to be used for contractile function *via* the creatine (Cr) kinase energy shuttle, in which phosphate is transferred from ATP to Cr with the formation of phosphocreatine (PCr) and ADP in a reaction catalyzed by mitochondrial Cr kinase (1): 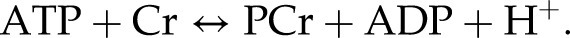


The Cr kinase system acts to keep ATP levels constant *via* a fall in PCr and a rise in cytosolic free ADP concentrations ([ADP]_free_), which controls mitochondrial oxidative phosphorylation when oxygen is not limiting (4).

In hypoxia, when oxygen availability limits oxidative phosphorylation, the heart moves toward more oxygen-efficient carbohydrate, away from fatty acid, metabolism (5). Thus, cardiac glucose uptake was increased in hypoxic rats (6) and in high-altitude-adapted humans (7), and fatty acid metabolizing enzyme [carnitine palmitoyltransferase 1 (CPT1) and medium-chain acyl-coenzyme A dehydrogenase (MCAD)] expression was decreased by hypoxia (8–10). Magnetic resonance (MR) studies have shown that cardiac PCr/ATP ratios are lower in high-altitude-adapted humans (11) and in lowlanders returning to sea level following a trek to Mount Everest Base Camp (12). However, the links among limited oxygen availability, changes in substrate metabolism, ATP generation, and cardiac function have not been well defined.

The balance between fatty acid and glucose metabolism may be regulated, at least in part, by the nuclear PPARs. Of the 3 receptor isoforms, α, δ, and γ, PPARα and PPARδ are highly expressed in the heart (13). PPARα regulates several genes encoding for proteins that control fatty acid metabolism, including MCAD (14), and glucose metabolism, including pyruvate dehydrogenase kinase 4 (PDK4) and glucose transporter 4 (GLUT4) (15).

Hypoxia is a potential driver of metabolic reprogramming, with the oxygen-sensitive transcriptional activator hypoxia-inducible factor 1α (HIF-1α) elevated in ischemic cardiac myocytes (16) and in infarcted hearts (17). In normoxia, HIF-α subunits are polyubiquitinated for proteasomal degradation, the constant degradation of HIF-α subunits mediated by hydroxylation *via* the prolyl hydroxylase domain (PHD) or egl-9 family hypoxia-inducible factor (EGLN) oxygenase family. In hypoxia, PHD activity is reduced, stabilizing HIF-α, which translocates to the nucleus and dimerizes with HIF-1β. The dimer transcriptionally activates ∼200 genes, including those involved in erythropoiesis, angiogenesis, and energy metabolism (18). In hypoxia-adapted Tibetans, variants of the genes *EGLN1* and endothelial PAS domain-containing protein 1, encoding for PHD2 (the most important of the 3 human PHDs) and the HIF-2α subunit, respectively, associated with the *PPARA* gene in genome-wide scans (19), and serum fatty acid concentrations in this group correlated with the PPARA haplotype (20). These findings suggest that PPARα may control cardiac substrate metabolism, ATP generation, and thereby, cardiac function in hypoxia (**Fig. 1**).

**Figure 1.**
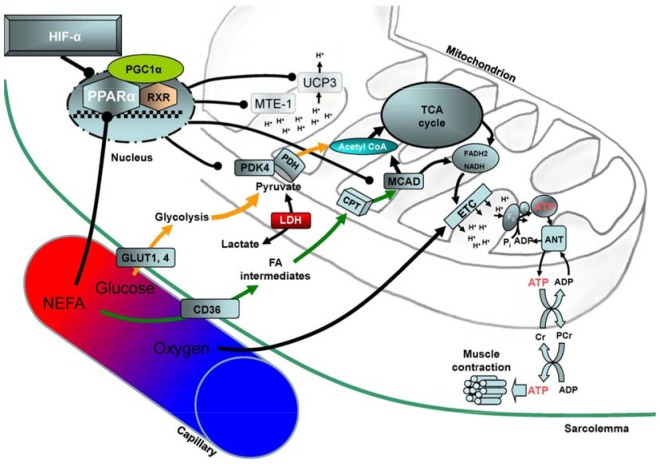
Putative mechanism for the control of cardiac substrate metabolism and function in hypoxia. Decreased oxygen tension in the blood inhibits PHD activity, stabilizing HIF-α subunits and down-regulating the nuclear hormone receptor, PPARα. In turn, PPARα regulates fatty acid and glucose metabolism *via* changes in several proteins, including MCAD and PDK4, and the mitochondrial inner membrane potential, *via* UCP3 and mitochondrial thioesterase 1 (MTE-1) proteins. Increased glycolytic flux and decreased mitochondrial uncoupling increase the efficiency of ATP production, the ATP being consumed primarily by myocardial contraction. ANT, adenine nucleotide translocase; CoA, coenzyme A; ETC, electron transport chain; FA, fatty acid; FADH2, flavin adenine dinucleotide; LDH, lactate dehydrogenase; NEFA, nonesterified fatty acid; PDH, pyruvate dehydrogenase; PGC-1α, PPARγ coactivator α; P_i_, inorganic phosphate; RXR, retinoid X receptor; TCA, tricarboxylic acid.

Recent studies using cultured cells, knockout, or transgenic mice seemingly demonstrate that alterations in the HIF system, whether decreases or increases in aryl hydrocarbon nuclear translocator (ARNT; HIF-1β) (21), HIF-1α (22–24), HIF-2α (21, 25), PHD1 (25), or von Hippel-Lindau (26) levels, increase PPARα or PPARγ (22) in heart or skeletal muscle. Such apparently contradictory studies necessitate a deeper understanding of the cellular mechanisms whereby a decrease in PPARα is absolutely required to maintain ATP levels for the contractile function of the hypoxic heart. Here, we show that hypoxia causes the heart to undergo a series of metabolic changes, coordinated by PPARα and designed to use the limited available oxygen as efficiently as possible. We demonstrate in normal mice how a high-fat diet increases PPARα expression, which prevents the metabolic changes required for acclimation to hypoxia, thereby significantly decreasing [ATP] and myocardial contractile function. We also demonstrate that the lack of myocardial PPARα prevents any metabolic response to hypoxia and that chemical induction of HIF, by inhibition of HIF hydroxylases in mechanically active cardiomyocytes, has the same effect on PPARα as hypoxia. In short, for adaptation to hypoxia, it is essential that PPARα expression decreases to increase the efficiency of oxygen use, *via* orchestrated changes in substrate metabolism and mitochondrial respiration, so that normoxic [ATP] and cardiac function can be maintained.

## MATERIALS AND METHODS

### Animals

Male wild-type 129Ev/Sv (*n* = 106; 14 mo) and littermate PPARα^−/−^ (*n* = 72) mice were housed on a 12 h light/dark cycle and fed *ad libitum*. The mice, a kind gift from Dr. Frank J. Gonzalez (National Institutes of Health, National Cancer Institute, Bethesda, MD, USA), were bred in-house on a pure 129Ev/Sv background with 10 backcrosses. All procedures conformed to ethical regulations of the United Kingdom Home Office.

### Hypoxia

Mice were housed in a glass-fronted hypoxia chamber (BioSpherix, Parish, NY, USA), in which N_2_ replaced O_2_. Chamber O_2_, monitored continuously, was used to regulate chamber N_2_ levels *via* a feedback system. Mice were fed laboratory chow (7.5% fat) or a high-fat (55% fat) diet. To acclimatize mice to hypoxia, chamber O_2_ was reduced in daily steps over a 7 d period to be maintained at 11% (v:v) for a further 12 d. Following hypoxia, animals were reoxygenated for 1 h to ameliorate any short-term effects of reoxygenation on cardiac function.

### Cardiac MRI

MRI was carried out on an 11.7 T (500 MHz) vertical bore (123-mm diameter) magnet (Magnex Scientific, Oxon, United Kingdom), as previously described (27).

### Heart perfusion

Mice were anesthetized using 60 mg ⋅ kg body weight^−1^ sodium pentobarbitone intraperitoneally and hearts were excised and arrested in ice-cold Krebs-Henseleit buffer. Hearts were perfused in Langendorff mode at 80 mmHg perfusion pressure and at 37°C with modified Krebs-Henseleit recirculating buffer gassed with 95% O_2_/5% CO_2_ containing 11 mM glucose and 0.4 mM palmitate prebound to 1.5% (w/v) albumin. A polyethylene balloon connected to a pressure transducer was inserted in the left ventricle (LV) and inflated to an end-diastolic pressure of 4–8 mmHg to measure pressures and heart rates.

### Cardiac substrate metabolism

Glycolytic flux and palmitate oxidation were measured in perfused hearts using 25 µCi [5-^3^H]-glucose or [9,10-^3^H]-palmitate, respectively, in recirculating perfusion buffer, as previously described (3, 28). Buffer samples were taken every 5 min for the measurement of [^3^H] label conversion to ^3^H_2_O using Dowex anion separation or Folch extraction. In a subset of experiments, insulin (500 μU) was added to the buffer to measure the maximal insulin-stimulated response. Hearts were snap frozen in liquid N_2_. Cardiac lactate efflux was determined by measuring lactate concentrations, using lactate dehydrogenase, in timed buffer collections.

### Cardiac high-energy phosphate metabolism

Perfused hearts were inserted into the same NMR system as used for imaging (see Cardiac MRI section), but using a 10 mm diameter probe (Rapid Biomedical, Wuerzburg, Germany). Fully relaxed spectra were acquired by averaging 60 scans using a 90° pulse (60 μs) and a 10-s delay to give an acquisition time of 10 min. Data were analyzed using jMRUI software and 4 spectra were summed to determine ATP, PCr, and P_i_ peak areas. Intracellular pH, the cytosolic-free ADP ([ADP]_free_), and the free energy of ATP hydrolysis (ΔG_ATP_) were calculated as previously described (4).

### HL-1 cardiomyocyte culture

HL-1 murine cardiomyocytes, a kind gift from Dr. William C. Claycomb (Louisiana State University Medical Center, New Orleans, LA, USA), were maintained in Claycomb Medium (Sigma-Aldrich, Dorset, United Kingdom). Cells were supplemented with 10% fetal bovine serum, ascorbic acid (0.3 mM), norepinephrine (0.1 mM), and l-glutamine (4 mM). Cells were cultured on plates precoated with 5 μg/ml fibronectin and 0.02% gelatin and incubated in 5% CO_2_ at 37°C and 95% humidity. All experiments were performed on confluent, beating cells. Cells were exposed to 2% hypoxia or normoxia for 24 h. In separate experiments, normoxic HL-1 cells were incubated for 24 h with a specific PHD inhibitor, 50 µM 2-(1-chloro-4-hydroxyisoquinoline-3-carboxamido) acetic acid (IOX3) (29, 30), which was synthesized specifically for the experiment (31). Cells were aspirated with PBS and harvested for protein analysis or quantitative RT-PCR (qRT-PCR).

### qRT-PCR analysis of cardiac and skeletal muscle and HL-1 cells

Primers were designed using Primer3 software based on GenBank or EnsemblGenomes browser searches and obtained from Eurofins MWG Operon (Wolverhampton, United Kingdom). References to primer sequences included the following: PPARα, NM_011144.6; PPARβ/δ, NM_011145.3; PPARγ, NM_011146.3; MCAD, NM_007382.4; UCP3, NM_009464.3; PDK4, NM_013743.2; VEGF, NM_009505.4; β-actin, NM_007393.3. Total RNA was extracted and purified from frozen heart and skeletal muscle tissues using a QiaAmp DNA Mini Kit (Qiagen, West Sussex, United Kingdom). For cells, total RNA was extracted and purified from ≤5 × 10^6^ confluent HL-1 cells using a QiaAmp kit (Qiagen). cDNA was synthesized from the RNA template using a high-capacity transcriptase kit (Thermo Fisher Scientific Life Sciences, Waltham, MA, USA). Quantitative PCR amplification was performed using the Real-Time PCR System (Thermo Fisher Scientific Life Sciences). The PCR program had an initial heat-activation step of 95°C for 10 min. Forty cycles of thermocycling were performed with a denaturation step at 95°C for 15 s and an annealing and extension step at 60°C for 1 min. Fluorescence was measured following each extension step. After amplification, a melting curve was acquired and used to determine the PCR product specificity. Relative quantification of target gene expression was normalized to the housekeeping gene, β-actin, and performed using a 2^−∆∆^*^Ct^* method.

### Tissue and cell lysate preparation and immunoblotting

Powdered frozen cardiac or skeletal muscle was added to 300 µl lysis buffer and homogenized for 30 s, as previously described (3, 28). Confluent HL-1 cardiomyocytes in 6-well plates were washed with PBS and added to 150 µl lysis buffer containing protease inhibitor. Lysates were boiled for 5 min and centrifuged at 13,000 rpm for 10 min, supernatant was saved, and protein concentrations were determined using a bicinchoninic acid protein assay kit (Fisher Scientific, Loughborough, United Kingdom). Samples were stored at −80°C following addition of 5% 2-ME (v/v) and boiling for 5 min.

Equal concentrations of protein from cardiac and skeletal muscle samples were loaded onto 12% SDS-PAGE gels, separated using electrophoresis, and transferred onto Immobilon-P membranes (Millipore, Watford, United Kingdom). Cardiac and skeletal muscle proteins were detected using the following polyclonal antibodies and dilutions in 5% milk: PGC-1α and MCAD (Santa Cruz Biotechnology, Dallas, TX, USA), 1:500; GLUT1 (Abcam, Cambridge, United Kingdom), 1:1000; GLUT4 (kind gift of Geoffrey Holman, University of Bath, United Kingdom), 1:4000; PDK4 and UCP3 (Abgent, Maidenhead, United Kingdom), 1:500 and 1:2500; MTE-1 (kind gift of Dr. Stefan Alexson, Karolinska Institute, Stockholm, Sweden), 1:2000; RXRα (Santa Cruz Biotechnology, London, United Kingdom), 1:1000; HIF-1 and -2α (Novus Biologicals, Littleton, CO, USA), 1:2000 and 1:500.

Secondary antibodies were horseradish peroxidase conjugate polyclonal with goat anti-rabbit specificity (Autogen-Bioclear, Wiltshire, United Kingdom), all diluted to 1:3500, with the exception of GLUT1 and -4, HIF-1α (1:2000) and -2α (1:1000), and MCAD (donkey anti-goat). Consistent protein loading and transfer were confirmed by Ponceau staining and protein levels related to internal standards to ensure homogeneity between samples and gels. Bands were quantified using UN-SCAN-IT software (Silk Scientific, Orem, UT, USA), and all samples were run in duplicate on separate gels to confirm results.

### Glycolytic enzyme activities

The myocardial enzymatic activities of 3-phosphoglycerate kinase (PGK) and pyruvate kinase (PK) were measured using coupled enzyme assays (32). Frozen, ground heart tissue (1 mg/ml) was extracted with the buffer containing 150 mM NaCl, 60 mM Tris-HCl, 5 mM EDTA, 0.2% Triton-X 100, 1 mM PMSF, 10 μg/ml leupeptin, 1 μg/ml aprotinin, pH 7.5. The PK activity was assayed at 30°C, 340 nm in medium containing 50 mM imidazole (pH 7.6), 20 mM KCl, 2 mM MgCl_2_, 0.1 mM EDTA, 0.1 mM NADH, 1 mM ADP, 4.5 U/ml lactate dehydrogenase, and 1 mM phosphoenolpyruvate. The PGK activity was recorded at 25°C, 340 nm, in an assay medium containing 50 mM imidazole buffer (pH 7.6), 2 mM MgCl_2_, 0.1 mM EDTA, 1 mM ATP, 5 mM 3-phosphoglycerate, and 0.2 mM NADH.

### Myocardial triacylglycerol assay

Cardiac lipids were extracted from freeze-clamped tissue (25–50 mg) using 10 ml 2:1 chloroform:methanol solution as previously described (3, 28). After mixing and phase separation, the lower phase was air dried at 50°C and resuspended in ethanol. With the use of a kit (Randox Laboratories, London, United Kingdom), cardiac triacylglycerol (TAG) content was measured spectrophotometrically at 500 nm.

### Statistics

Grouped data (means ± sem) were analyzed using 3-factor ANOVAs, with individual comparisons subsequently performed using Student's *t* tests. Results were considered significantly different at *P* < 0.05.

## RESULTS

### Effects of hypoxia on mouse physiology and cardiac morphology

**Figure 2.**
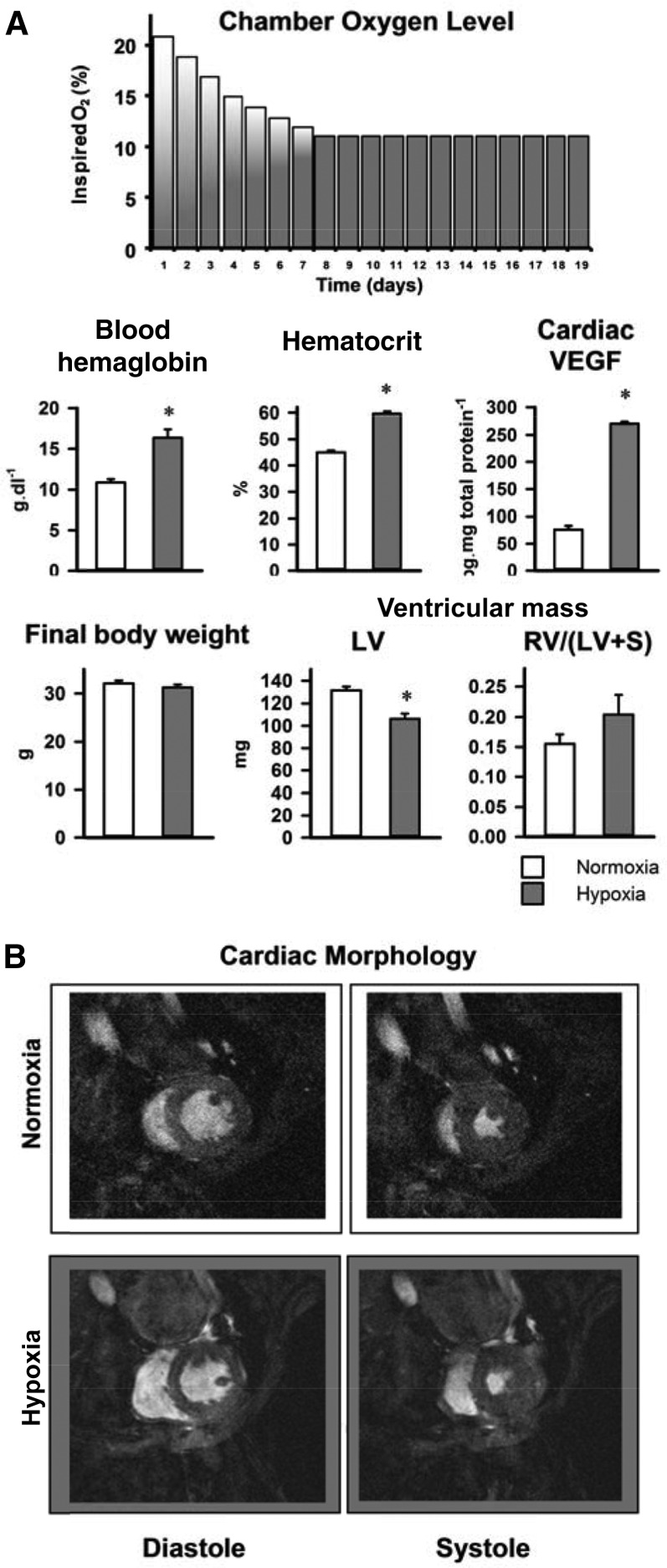
Effect of 3 wk hypoxia on whole-body physiology (*A*) and *in vivo* cardiac morphology (*B*) in wild-type mice fed a chow diet; *n* = 6. S, septum. *P < 0.05 *vs*. normoxic chow-fed control.

To investigate functional and metabolic responses of the *in vivo* mouse heart to hypoxia, we used a 3-wk protocol that began with 7 d of lowering ambient oxygen concentration from 21 to 11% in daily increments to produce a graded, physiologic hypoxia (**Fig. 2**). Hypoxia in wild-type, chow-fed mice increased whole-blood hemoglobin by 51% (*P* < 0.001) and hematocrit by 33% (*P* < 0.001) but did not alter body weight. Established HIF-1α downstream targets, cardiac VEGF and PDK1 protein, increased 3.6-fold (*P* < 0.001) and 1.8-fold (*P* < 0.01), respectively. LV mass decreased by 19% (*P* < 0.001) following hypoxia, whereas right-ventricular (RV) mass was unaltered. Plasma metabolites in wild-type mice were unchanged by hypoxia (Supplemental Fig. 1).

### Effects of hypoxia on cardiac function and metabolism in chow-fed, wild-type mice

**Figure 3.**
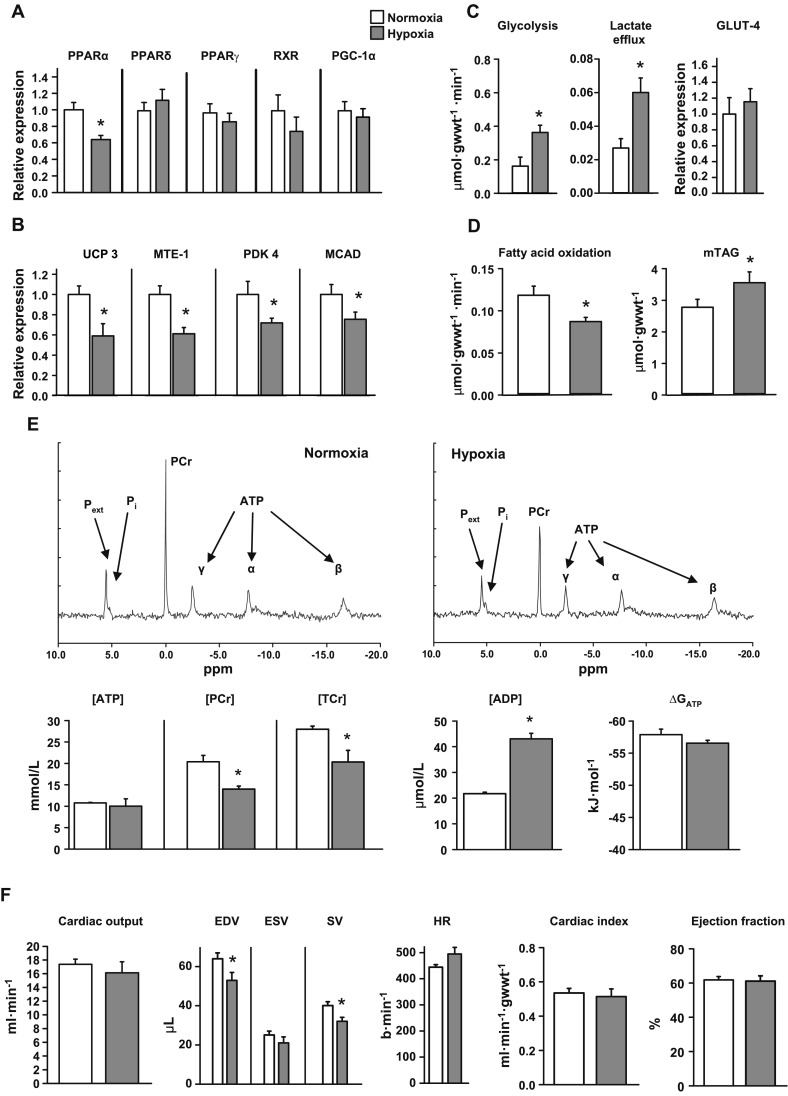
Effect of chronic hypoxia on cardiac metabolism and function. Effects of 3 wk of hypoxia on cardiac PPARα and PPARδ mRNA, plus PPARγ, RXR, and PGC-1α proteins (*A*), PPARα-controlled proteins (*B*), myocardial carbohydrate and lipid metabolism (*C*, *D*), [^31^P] MR spectra and high-energy phosphate metabolism (*E*), and *in vivo* cardiac function (*F*) in wild-type mice fed a (7.5% fat) chow diet; min *n* = 6. *P < 0.05 *vs*. normoxic chow-fed control; minimum *n* = 6. gwwt, gram wet weight; mTAG, intramuscular TAG; P_ext_, extracellular P_i_; TCr, total Cr; ESV, end-systolic volume; b, beats; HR, heart rate.

Hypoxia in chow-fed, wild-type mice decreased cardiac PPARα mRNA levels by 36% (*P* < 0.05), with PPARδ mRNA and PPARγ, RXR, and PGC-1α protein levels remaining unchanged (**Fig. 3*A*** and Supplemental Fig. 2). In parallel with lower PPARα mRNA levels, hypoxia decreased protein levels of downstream targets of PPARα: UCP3, MTE-1, and PDK4 (Fig. 3*B*). MCAD protein levels were 24% (*P* < 0.05) lower, but proteins involved in mitochondrial respiration were unchanged following hypoxia (Supplemental Fig. 2).

Hypoxia increased cardiac glycolysis 2.3-fold (*P* < 0.01) and lactate efflux 2.4-fold (*P* < 0.01), measured in isolated, beating, perfused hearts using [^3^H] substrate labeling (Fig. 3*C*). Hypoxia did not alter total cardiac GLUT4 protein or activities of glycolytic intermediates (Supplemental Fig. 3). Fatty acid oxidation was 26% (*P* < 0.02) lower in hearts from hypoxic mice compared with normoxic controls (Fig. 3*D*), whereas myocardial TAG levels were 28% (*P* < 0.05) higher in hypoxic mice. In isolated, perfused hearts, [PCr] was 31% (*P* < 0.001) lower, but [ATP] was unaltered by hypoxia, measured using [^31^P] NMR spectroscopy and HPLC (Fig. 3*E*); total Cr was 28% (*P* < 0.05) lower following hypoxia. Although [ADP]_free_ increased 2-fold (*P* < 0.02), the calculated ΔG_ATP_ was preserved. *In vivo* cine MRI showed that 3 wk of hypoxia did not alter cardiac output, cardiac index, or ejection fraction, despite an 18% (*P* < 0.02) lower stroke volume (SV), resulting from a 17% (*P* < 0.05) lower end-diastolic volume (EDV) in hypoxic mice (Fig. 3*F*).

### Effect of hypoxia on cardiac function and metabolism in high-fat–fed, wild-type mice

**Figure 4.**
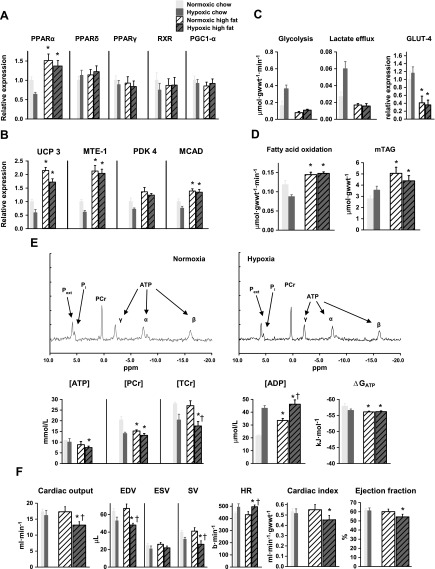
Effect of chronic hypoxia on cardiac metabolism and fuction in high-fat fed mice. Effects of a high-fat (55% fat) diet plus 3 wk of hypoxia in wild-type mice on cardiac PPARα and PPARδ mRNA, plus PPARγ, RXR, and PGC-1α proteins (*A*), PPARα controlled proteins (*B*), myocardial carbohydrate and lipid metabolism (*C*, *D*), [^31^P] MR spectra and high-energy phosphate metabolism (*E*), and *in vivo* cardiac function (*F*); minimum *n* = 6. Data in gray show hypoxic response of chow-fed, wild-type mice for comparison. *P < 0.05 *vs*. normoxic chow-fed control; ^†^*P* < 0.05 *vs*. normoxic, high-fat–fed control.

The cardiac metabolic and energetic changes observed in hypoxic mice may have been caused by decreased PPARα expression. To investigate whether PPARα expression is central to hypoxic adaptation, we prevented PPARα down-regulation by increasing PPARα expression using high-fat (55% fat) feeding. The feeding of mice a high-fat diet for 3 wk in normoxia did not alter body weight, but with hypoxia, caused a 10% (*P* < 0.01) loss of body weight and a 19% (*P* < 0.01) decrease in LV mass (Supplemental Table 1). High-fat feeding increased PPARα mRNA expression in both normoxia and hypoxia (*P* < 0.05), preventing any hypoxia-induced decrease (**Fig. 4*A***). PPARδ mRNA and PPARγ, RXR, and PGC-1α protein levels were unchanged by high-fat feeding. Downstream targets of PPARα, UCP3, and MTE-1 proteins, increased 2-fold (*P* < 0.001), and MCAD protein increased by 39% (*P* < 0.01) with high-fat feeding under both normoxia and hypoxia (Fig. 4*B* and Supplemental Fig. 2). High-fat feeding did not significantly increase cardiac PDK4 protein, although it prevented the decrease caused by hypoxia in chow-fed mice.

High-fat feeding prevented the increase in cardiac glycolysis and lactate efflux that occurred in chow-fed mice in hypoxia, and decreased GLUT4 protein by 59% (*P* < 0.05), which remained unaltered by hypoxia (Fig. 4*C*). High-fat feeding increased cardiac fatty acid oxidation by 22% (*P* < 0.05; Fig. 4*D*) and myocardial TAG content by 82% (*P* < 0.01), both unaffected by hypoxia. High-fat feeding decreased cardiac [PCr] by 25% (*P* < 0.05) in normoxic mice, but [PCr] did not decrease further with hypoxia (Fig. 4*E*). [ATP] was normal in normoxic high-fat–fed mouse hearts but was 31% (*P* < 0.05) lower in hearts from hypoxic high-fat–fed mice compared with chow-fed normoxic mice. Total Cr was unaltered in normoxic high-fat–fed hearts but was 37% (*P* < 0.05) lower in hearts from high-fat–fed mice exposed to hypoxia. [ADP]_free_ was 55% (*P* < 0.05) higher in hearts from normoxic high-fat–fed mice compared with normoxic chow-fed mice and increased by a further 38% with hypoxia (*P* < 0.05). High-fat feeding significantly lowered cardiac ΔG_ATP_ in all mouse hearts.

High-fat feeding under normoxia had no effect on cardiac function (Fig. 4*F*), whereas feeding a high-fat diet under hypoxia decreased *in vivo* cardiac output by 24% compared with normoxic chow (*P* < 0.01) or high-fat-fed (*P* < 0.05) mice. This was a result of a 35% (*P* < 0.001) lower SV, accounted for by a 28% (*P* < 0.01) lower EDV and despite a significantly higher heart rate. The cardiac index was decreased by 15% (*P* < 0.05), and ejection fractions were 12% (*P* < 0.02) lower than in normoxic chow-fed mice. Hence, high-fat feeding prevented the adaptive molecular and metabolic responses to hypoxia that were observed in chow-fed mouse hearts, thereby causing cardiac dysfunction.

### Effect of hypoxia on cardiac function and metabolism in PPARα^−/−^ mice

**Figure 5.**
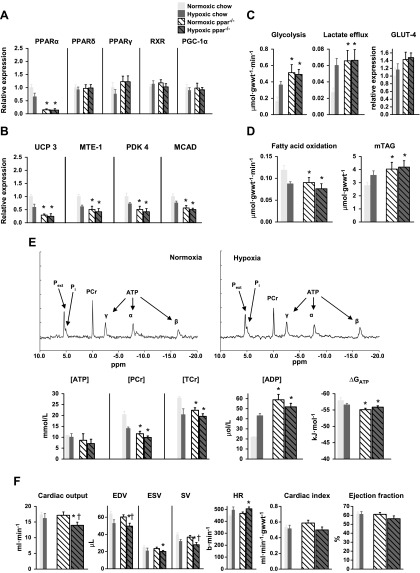
Effects of 3 wk of hypoxia in PPARα^−/−^ mice, fed a chow (7.5% fat) diet, on cardiac PPARα and PPARδ mRNA, plus PPARγ, RXR, and PGC-1α proteins (*A*), PPARα controlled proteins (*B*), myocardial carbohydrate and lipid metabolism (*C*, *D*), [^31^P] MR spectra and high-energy phosphate metabolism (*E*), and *in vivo* cardiac function (*F*); minimum *n* = 6. Data in gray show the hypoxic response of chow-fed, wild-type mice for comparison. *P < 0.05 *vs*. normoxic chow-fed control; ^†^*P* < 0.05 *vs*. normoxic PPARα^−/−^ chow fed.

Having demonstrated that increased PPARα was associated with impaired function of the hypoxic heart, we next determined whether loss of PPARα altered cardiac metabolic and functional responses to hypoxia. PPARα^−/−^ mice were fed a chow diet and housed under hypoxia for 3 wk. PPARα^−/−^ mice had 13% (*P* < 0.05) lower body weight and 18% (*P* < 0.05) lower LV mass than wild-type mice, with neither altered by hypoxia (Supplemental Table 1). Cardiac PPARα mRNA levels in PPARα^−/−^ mice were negligible, although PPARδ mRNA and PPARγ, RXR, and PGC-1α protein levels were normal and unchanged by hypoxia (**Fig. 5*A*** and Supplemental Fig. 2). Cardiac UCP3 protein (Fig. 5*B*) was 71% (*P* < 0.001) lower, and MTE-1 was 49% (*P* < 0.02) lower in PPARα^−/−^ mouse hearts compared with wild-type mice, and both were unchanged by hypoxia. Other key mitochondrial proteins were unaltered relative to wild-type mice (Supplemental Fig. 2). PDK4 was 45% lower (*P* < 0.01), and MCAD protein was 43% (*P* < 0.001) lower in normoxic and hypoxic PPARα^−/−^ mouse hearts.

PPARα^−/−^ mouse hearts had 3-fold (*P* < 0.01) higher glycolytic rates than normoxic wild-type mouse hearts and were unaffected by hypoxia (Fig. 5*C*). Glycolysis was not maximal following hypoxia, as stimulation with insulin doubled glycolytic rates in both normoxic and hypoxic PPARα^−/−^ mouse hearts (Supplemental Fig. 4). Lactate efflux from hypoxic PPARα^−/−^ mouse hearts was 2-fold (*P* < 0.01) higher than from normoxic wild-type hearts and not different from normoxic PPARα^−/−^ hearts. Cardiac GLUT4 protein was similar in normoxic and hypoxic PPARα^−/−^ mice. Consistent with PPARα being a regulator of fatty acid metabolism, PPARα^−/−^ mice had 24% lower (*P* < 0.05) cardiac fatty acid oxidation (Fig. 5*D*) than wild-type mice, which was unaltered by hypoxia. Cardiac TAG content was increased by 45% (*P* < 0.05) in normoxic and hypoxic PPARα^−/−^ mouse hearts. Thus, the lack of PPARα prevented the metabolic changes that were observed in hypoxic wild-type mouse hearts.

In PPARα^−/−^ mice, [PCr] was decreased by 43% (*P* < 0.001), although [ATP] was unaltered compared with normoxic wild-type mouse hearts (Fig. 5*E*). Hypoxic cardiac [PCr] and [ATP] were decreased by 51% (*P* < 0.001) and 34% (*P* < 0.05), respectively, in PPARα^−/−^ mice. Total Cr was decreased by 20% (*P* < 0.001) in PPARα^−/−^ mouse hearts with no effect of hypoxia. As a consequence, [ADP]_free_ was 2.7-fold (*P* < 0.05) higher and ΔG_ATP_ significantly lower in all PPARα^−/−^ mouse hearts, and unaltered by hypoxia.

Normoxic PPARα^−/−^ mice had normal cardiac function, but hypoxic PPARα^−/−^ mice had 23% lower EDV (*P* < 0.01), 19% lower ESV (*P* < 0.05), 29% (*P* < 0.01) lower SV, and therefore, 20% lower cardiac output than normoxic wild-type mice (Fig. 5*F*). Therefore, deletion of PPARα significantly altered myocardial substrate and energy metabolism and prevented any metabolic response to hypoxia, thereby impairing cardiac function.

### Effect of hypoxia on cardiac function and metabolism in PPARα^−/−^ mice fed a high-fat diet

**Figure 6.**
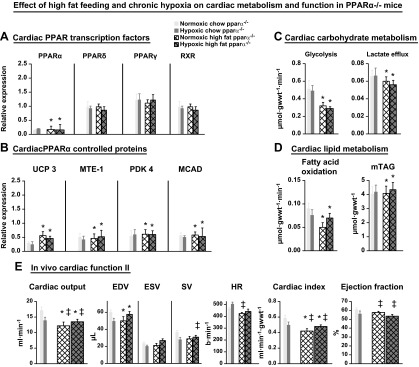
Effects of 3 wk of hypoxia on PPARα^−/−^ mice fed a high-fat (55% fat) diet on cardiac PPARα and PPARδ mRNA, plus PPARγ and RXR proteins (*A*), PPARα controlled proteins (*B*), myocardial carbohydrate and lipid metabolism (*C*, *D*), and *in vivo* cardiac function (*E*); minimum *n* = 6. Data in gray show the hypoxic response of chow-fed PPARα^−/−^ mice for comparison. *P < 0.05 *vs*. chow-fed control (not shown); ^‡^*P* < 0.05 *vs*. chow-fed PPARα^−/−^.

To test whether high-fat feeding was operating *via* an alternative mechanism to PPARα regulation, PPARα^−/−^ mice were fed a high-fat diet and housed in normoxia or hypoxia. High-fat feeding had no effect on body weight or LV mass compared with chow-fed PPARα^−/−^ mice (Supplemental Table 1). Cardiac PPARα and PPARδ mRNA levels and PPARγ and RXR protein concentrations were unaltered by high fat feeding, being the same as in the chow-fed PPARα^−/−^ mice, with no effect of hypoxia (**Fig. 6*A***). Cardiac UCP3, MTE-1, PDK4, and MCAD proteins were unaltered in PPARα^−/−^ mice fed a high-fat diet compared with a chow diet, again unaffected by chronic hypoxia (Fig. 6*B*). Cardiac metabolism in PPARα^−/−^ mice was also unaltered by either high-fat feeding with or without (Fig. 6*C*, *D*). Cardiac output in PPARα^−/−^ mice fed a high-fat diet was 29% lower than chow-fed PPARα^−/−^ mice (*P* < 0.01) and 18% lower with hypoxia (*P* < 0.01), similar to chow-fed PPARα^−/−^ mice exposed to chronic hypoxia (Fig. 6*E*). Cardiac index was 29% lower in PPARα^−/−^ mice fed a high-fat diet *vs*. chow, with no effect of hypoxia (*P* < 0.01). Ejection fraction was 6% lower in high-fat–fed PPARα^−/−^ mice and unaltered by hypoxia (*P* < 0.05). Therefore, PPARα ablation nullified the metabolic flexibility seen in healthy mice fed a high-fat diet, resulting in impaired cardiac function and unaffected by hypoxia.

### Effect of hypoxia and a high-fat diet on whole-body physiology and PPARα-regulated proteins in skeletal muscle

**Figure 7.**
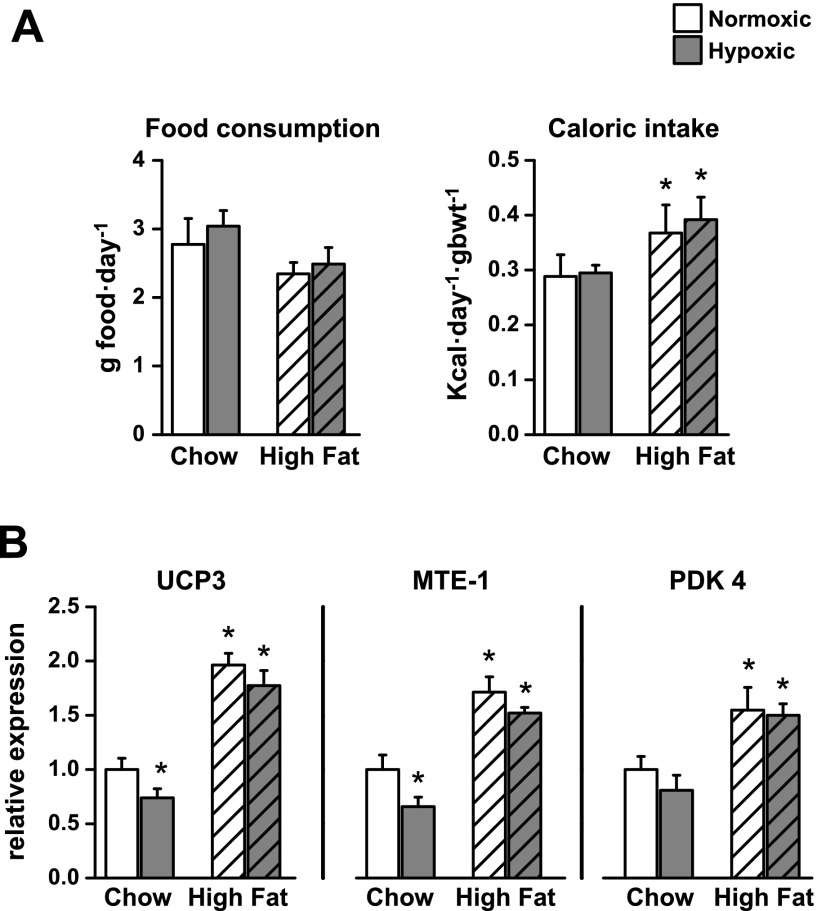
Effect of 3 wk of hypoxia on energy intake (*A*; *n* = 4) and skeletal muscle proteins (*B*; *n* = 6) under PPARα control in wild-type mice fed chow (7.5% fat) or a high-fat (55% fat) diet. *P < 0.05 *vs*. normoxic chow-fed control. gbwt, gram body weight.

To investigate the weight loss observed in wild-type hypoxic mice fed a high-fat diet (Supplemental Table 1), energy intake was measured during the 3-wk protocol. Food consumption was not affected by hypoxia or by the high-fat diet (**Fig. 7*A***). However, as a result of higher calorific value, there was a 28% (*P* < 0.05) higher energy intake on the high-fat diet, which again was unaffected by hypoxia. Cardiac levels of UCP3 and MTE-1 were elevated by high-fat feeding (Fig. 4*B*), suggesting that skeletal muscle UCPs responded to the high-fat diet in a similar manner, thereby contributing to body-weight loss in hypoxia. UCP3 protein levels in skeletal (gastrocnemius) muscle were 26% (*P* < 0.05) lower after 3 wk of hypoxia (Fig. 7*B* and Supplemental Fig. 5), a similar response to that in cardiac muscle (Fig. 3*B*). As found in cardiac muscle, UCP3 levels in skeletal muscle increased with high-fat feeding compared with chow-fed controls (*P* < 0.001) and were not altered by hypoxia. Skeletal muscle MTE-1 protein levels showed identical responses to those of UCP3, being 34% (*P* < 0.05) lower in hypoxic, chow-fed mice and 71% (*P* < 0.001) higher after high-fat feeding with/without hypoxia. PDK4 protein levels increased by 55% (*P* < 0.01) during high-fat feeding, which again prevented the adaptive responses to hypoxia.

### Effect of hypoxia or direct HIF activation on HL-1 cardiac cells

**Figure 8.**
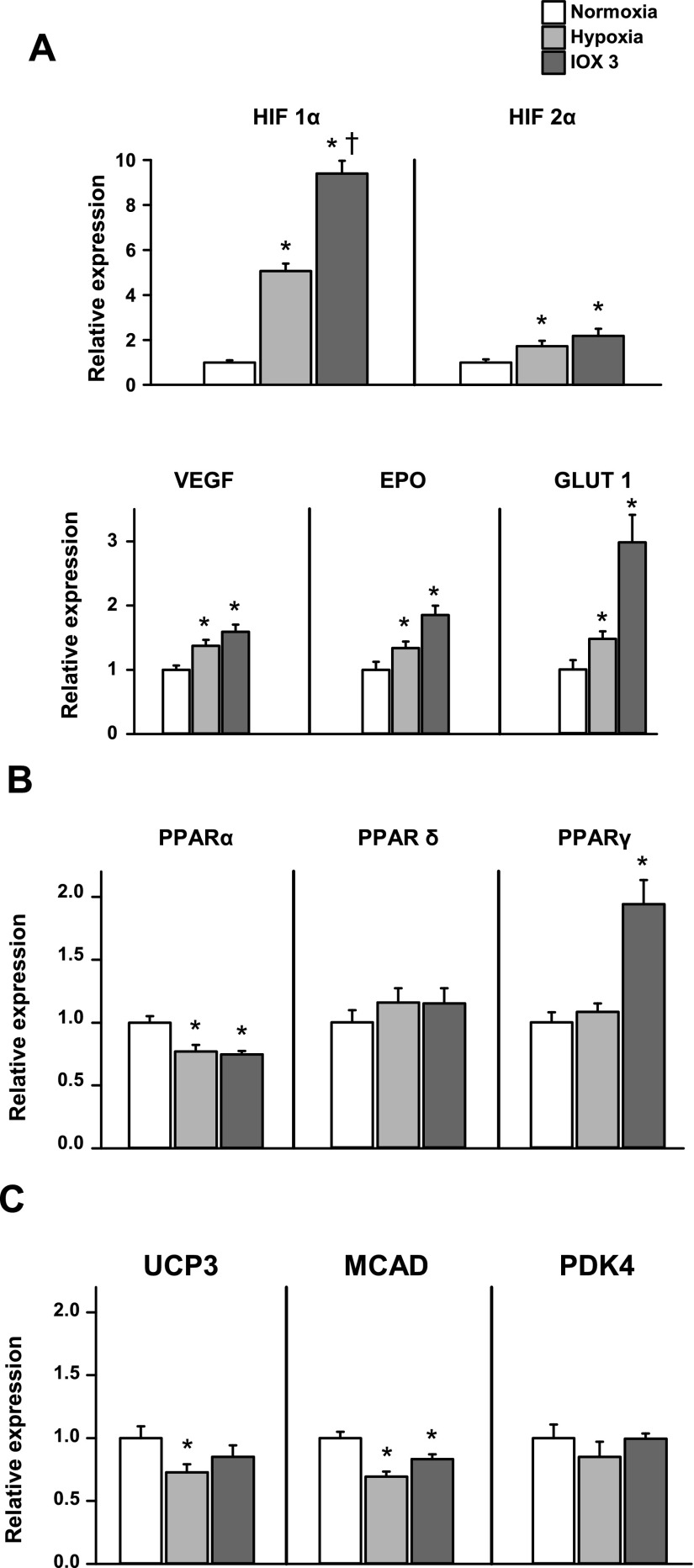
Effect of 24-h hypoxia or a PHD inhibitor, IOX3, on HL-1 cardiomyocytes; *n* = 4. *A*) Hypoxic cell markers. *B*) PPAR isoform expression. *C*) PPARα controlled mRNA expression. *P < 0.05 *vs*. normoxic cells; ^†^*P* < 0.05 *vs*. hypoxic cells. EPO, Erythropoietin.

Having established that adaptation to cardiac hypoxia was regulated by PPARα, we used confluent, beating HL-1 cardiac cells to investigate whether such adaptations were directly linked to the HIF system. Cells were exposed to hypoxia (2% O_2_) for 24 h, or the HIF system was chemically induced using 24 h incubation with the selective prolyl hydroxylase inhibitor, IOX3 (33). Hypoxia caused a 5-fold increase in HIF-1α (*P* < 0.001) and a 72% increase in HIF-2α (*P* < 0.05) protein levels (**Fig. 8*A*** and Supplemental Fig. 5). IOX3 was a more potent activator of the HIF system than hypoxia, with a 9-fold increase in HIF-1α (*P* < 0.001) and doubling of HIF-2α (*P* < 0.01) protein levels after 24 h incubation. Under HIF-1α control, VEGF mRNA increased by 37% (*P* < 0.05) in hypoxia and 59% (*P* < 0.001) with IOX3. PDK1 protein levels increased 34% in hypoxia (*P* < 0.05) and 86% with IOX3 treatment (*P* < 0.01). GLUT1 protein levels increased by 48% (*P* < 0.02) with hypoxia and 3-fold (*P* < 0.01) with IOX3.

Hypoxia decreased PPARα mRNA by 23% (*P* < 0.01) in HL-1 cells (Fig. 8*B*), similar to that caused by *in vivo* hypoxia in the intact heart. IOX3 produced a similar, 25% decrease in PPARα mRNA (*P* < 0.001). PPARδ mRNA levels were unaffected by hypoxia or IOX3. PPARγ mRNA levels were not altered by hypoxia but were doubled (*P* < 0.001) after IOX3 incubation.

In cultured cardiac cells, PPARα-controlled mRNA expression was altered by hypoxia in a similar manner as the respective protein in the *in vivo* hypoxic heart (Fig. 8*C*). UCP3 levels were lowered by 27% (*P* < 0.01) by hypoxia but were not altered by IOX3. MCAD levels were 31% (*P* < 0.001) lower after hypoxia and 17% (*P* < 0.01) lower after IOX3. PDK4 levels were unaffected by hypoxia or IOX3.

## DISCUSSION

In this study, we have demonstrated that normobaric hypoxia in mice decreased myocardial PPARα expression, fatty acid oxidation, and mitochondrial UCP3 and increased carbohydrate metabolism; such changes allowed normoxic [ATP] and contractile function to be maintained. Genetic ablation of PPARα in mice caused metabolic changes in the normoxic mouse heart that were already at the extreme of hypoxic adaptation and therefore, could not be altered by hypoxia. A high-fat diet increased mouse heart PPARα expression and prevented PPARα down-regulation by hypoxia. Thus, with high-fat feeding, the cardiac proteins under PPARα control (UCP3, MTE-1, PDK4, and MCAD) and fatty acid oxidation increased, whereas GLUT4 levels and glycolysis decreased; metabolic changes that remained in hypoxia. Consequently, the high-fat diet plus 3 wk exposure to hypoxia significantly decreased myocardial [ATP], cardiac index, and ejection fraction. To our knowledge, this is the first time that an increase in a dietary constituent, fat, has been shown to cause myocardial dysfunction in hypoxia, a finding that may have implications for altitude exposure (34) or heart failure (in which similar metabolic changes, the “fetal gene program,” occur) (1, 28, 35, 36). However, the human diet typically contains ∼30% fat and probably seldom the 7.5 or 55% fat used in the mouse diets.

PPARα down-regulation and substrate switching are essential for the maintenance of contractile function of the hypertrophied heart (37), and there have been reports of decreased PPARα expression in the heart following *in vivo* hypoxia (24, 26–28). In our mice, PPARα appeared to be the sole PPAR isoform involved in the myocardial metabolic changes, as neither PPARδ nor PPARγ levels changed. In agreement with findings in human skeletal muscle at altitude (38), there were no changes in PGC-1α nor were cardiac RXR protein levels decreased significantly by hypoxia. In cultured cardiomyocytes, however, others have demonstrated decreased hypoxia-induced PPARα/RXR DNA binding as a result of decreased RXR (39) or increased HIF-1α (24) levels. PPARα has been linked to hypoxic adaptation in high-altitude tolerant humans (19), and simulated hypoxia, using cobalt chloride to inhibit prolyl hydroxylases (40), decreased PPARα mRNA (9), suggesting regulation of PPARα *via* the HIF system. Certainly, in beating cardiomyocytes, we found that hypoxia or prolyl hydroxylase inhibition increased HIF-1α and HIF-2α, and decreased PPARα, MCAD, and UCP3 expression; beneficial adaptations to hypoxia (41).

Yet, studies of the HIF complex in transgenic (23) or knockout mouse hearts (21, 22, 26) or skeletal muscle (25) have yielded apparently conflicting (and confusing) results, in that decreased HIF-1β (21), HIF-1α (22, 23), or HIF-2α (21) or increased HIF-1α (26) or HIF-2α (25) are reported to increase (21, 23, 25) or not alter (22) PPARα or decrease PPARγ (22) expression with increased (21), or no change in (25), fatty acid oxidation. Of course, these are not studies on the metabolic effects of hypoxia, but on the contribution of individual constituents of the HIF pathway to changes in metabolism *via* the PPAR transcription factors. So, both increased (25) and decreased (21) HIF-2α increased PPARα expression, whereas decreased HIF-1α increased either PPARα (23) or PPARγ (22) expression. It is difficult to compare such studies, as few and rarely the same measures of myocardial metabolism or function have been made, and seldom using the same techniques.

However, most studies reported the deposition of lipids in the heart (21–23, 26) or cardiomyocytes (24), whether HIF-1α was increased (24, 26) or decreased (22, 23). We also found increased TAG in all hypoxic mouse hearts, whether PPARα levels and fatty acid oxidation were increased or decreased and unrelated to the effects of hypoxia on contractile function. Indeed, both the high-fat–fed and the PPARα^−/−^ mouse hearts had high TAG levels in normoxia that were unaltered by hypoxia, suggesting that more fatty acids were taken up than could be oxidized and that lipid deposits per se do not cause cardiac injury.

Carbohydrate metabolism was increased with hypoxia, but high-fat feeding prevented this potentially adaptive mechanism. GLUT4 was down-regulated with activation of PPARα *via* high-fat feeding and was unaltered by chronic hypoxia. Consistent with this finding, PPARα overexpression is known to repress GLUT4 mRNA (15), and we have shown that cardiac GLUT4 content is reduced in obesity (42). Lower GLUT4 levels may have contributed to cardiac dysfunction when hypoxia was combined with a high-fat diet, preventing increased carbohydrate metabolism and resulting in lower cardiac [ATP] in hypoxic mice.

Ventricular mass was lower in chronically hypoxic mice, resulting in cardiac hypotrophy. This reflects human studies of altitude exposure in sea-level natives (12). The stimulus for such reduction in LV mass, in the absence of loss of body weight, may be a requirement to improve oxygen diffusion distances in tissue with a sustained, high-oxygen demand (43). The molecular mechanisms associated with such loss of cardiac muscle are incompletely understood but may involve a reversal of the hypertrophic mechanisms associated with physiologic hypertrophy (44). In hypoxic mice fed a high-fat diet, whereas absolute LV mass fell in hypoxia, cardiac hypotrophy was not evident, as hypoxic high-fat–fed animals also lost body weight. The molecular mechanisms for this weight loss are unclear; however, all groups of PPARα^−/−^ mice in this study were smaller than wild-type littermates, which potentially implicates PPARα in the regulation of body weight.

Not only did hypoxia decrease PPARα mRNA levels and PPARα downstream targets—UCP3, MTE-1, and MCAD protein levels—in the heart, but also in skeletal muscle. In contrast to the increased PPARα observed in skeletal muscle in the PHD1^−/−^ mouse (25), the decrease in PPARα expression is in agreement with findings in human skeletal muscle at an altitude in which UCP3 levels and β-hydroxyacyl-coenzyme A dehydrogenase, a β-oxidation enzyme and PPARα target, were down-regulated (38). The adaptation to hypoxia *via* changes in PPARα expression, observed here in heart and skeletal muscle, may be relevant to other organs that have high PPARα expression, such as liver, kidney, and intestine (45). As in the heart, we found that the high-fat diet increased (and prevented any hypoxia-induced decrease in) UCP3, MTE-1, and PDK4 in mouse skeletal muscle. Although the high-fat–fed mice consumed 28% more calories than those on the chow diet, they did not gain weight, possibly owing to energy wastage *via* increased mitochondrial uncoupling (3).

The normobaric, hypoxia-induced decrease in cardiac PCr in our mice has been shown in the human heart as a decrease in PCr/ATP, measured using *in vivo* MRS: in lowlanders following a trek to Mount Everest Base Camp (12) and in Sherpa volunteers, even after 4 wk of de-acclimation at low altitudes (11). The decreased cardiac PCr/ATP in the Sherpa hearts was attributed to the requirement for a higher [ADP]_free_, to be closer to the Km of 110 μM (46) for ADP-requiring PK activity in glycolysis, reflecting the elevated contribution of carbohydrate-to-myocardial energy needs (7, 11). Such a mechanism may be related to a decrease in PPARα levels *via* high-altitude adaptation of the *PPARA* gene in Tibetans (19) and could explain why the highest [ADP]_free_ occurred in the highly glycolytic PPARα^−/−^ mouse hearts in normoxia and hypoxia. However, a genetic adaptation cannot explain the 18% decrease in myocardial PCr/ATP and diastolic dysfunction in human lowlanders after a trek to Mount Everest (12), which may have been exacerbated by the hypobaria and a result of the requirement for increased [ADP]_free_, plus the insulin resistance and elevated epinephrine observed in the same cohort (47).

[ADP]_free_ is a key metabolite controlling oxidative phosphorylation (4), so it is possible that the decrease in PCr in the hypoxic mouse hearts was a result of the need for higher [ADP]_free_ to stimulate mitochondrial respiration and to increase glycolytic flux. The requirement for oxygen would have been decreased in the hypoxic hearts by the decrease in both fatty acid oxidation and mitochondrial uncoupling, with increased flux through pyruvate dehydrogenase and increased glucose oxidation, all of which allowed [ATP] and contractile function to remain at normoxic levels.

In the mice fed a high-fat diet in normoxia, myocardial PCr decreased with no loss of total Cr to maintain normal ATP levels and cardiac function despite the increased oxygen required for fatty acid oxidation and increased oxygen wastage *via* mitochondrial uncoupling. Such changes with high-fat diets have been observed in human (48) and rat hearts associated with decreased efficiency (3). However, when the oxygen supply was restricted, the combination of greater oxygen requirement for fatty acid oxidation, plus oxygen wastage via increased mitochondrial UCP3 proteins, significantly decreased ATP, cardiac index, and ejection fraction. Likewise, mice with a cardiac-specific ARNT ablation had increased PPARα expression and fatty acid oxidation with decreased ejection fraction (21).

The metabolic changes caused by hypoxia in wild-type mouse hearts were found to have occurred already in normoxic PPARα^−/−^ mouse hearts and did not change further with hypoxia. PPARα^−/−^ mouse hearts maintained normal cardiac index and ejection fractions in hypoxia, owing to high [ADP]_free_, high glycolytic flux, low PDK4 (suggesting increased pyruvate dehydrogenase flux and glucose oxidation), low fatty acid oxidation, and low mitochondrial UCP3 and MTE-1 levels, all of which would have resulted in the most efficient use of any available oxygen. Thus, metabolism in the normoxic PPARα^−/−^ mouse heart may be viewed as at the extreme of hypoxic adaptation, which increases resistance to ischemia (49, 50), but may not support a high workload (51) with greater hypertrophy and functional impairment following aortic constriction (52). It is possible that the PPARα^−/−^ mouse hearts may have had decreased ejection fractions, in addition to the lower cardiac output and higher heart rates, with a longer duration of hypoxia. As metabolism in PPARα^−/−^ mouse hearts was at the limit of hypoxic adaptation during normoxia, with no response to hypoxia, the use of PPARα^−/−^ mice to validate PPARα as a HIF target is questionable. For example, knockout mice with decreased HIF-2α (HIF-1β^−/−^) (21), increased HIF-2α (Phd1^−/−^) (25), and decreased HIF-1α [NADPH oxidase deficient (Tg Nox^−/−^) transgenic mice] (22) had their metabolic phenotypes reversed in a double knockout made by crossing with PPARα^−/−^ mice.

In summary, we propose that decreased myocardial PPARα expression is central to the metabolic changes required to maintain contractile function in hypoxia. A high-fat diet increased PPARα expression, fatty acid oxidation, and mitochondrial UCP levels—changes that were tolerated in normoxia, but decreased ATP and impaired cardiac function in hypoxia. Finally, the metabolic changes in normoxic PPARα^−/−^ mouse heart can be viewed as at the extreme of hypoxic metabolic adaptation, in that they have no metabolic response to hypoxia.

## Abbreviations

ΔG_ATP_: free energy of ATP hydrolysis, [ADP]_free_, cytosolic-free ADP concentration
ARNT: aryl hydrocarbon nuclear translocator
CPT: carnitine palmitoyltransferase
Cr: creatine
EDV: end-diastolic volume
EGLN: egl-9 family hypoxia-inducible factor
ESV: end-systolic volume
FFA: free fatty acid
GLUT: glucose transporter
HIF: hypoxia-inducible factor
IOX3: 2-(1-chloro-4-hydroxyisoquinoline-3-carboxamido) acetic acid
LV: left ventricular
MCAD: medium-chain acyl-coenzyme A dehydrogenase
MR: magnetic resonance
MRS: magnetic resonance spectroscopy
MTE-1: mitochondrial thioesterase 1
PCr: phosphocreatine
PDK: pyruvate dehydrogenase kinase
PGC-1α: peroxisome proliferator-activated receptor-γ coactivator α
PGK: 3-phosphoglycerate kinase
PHD: prolyl hydroxylase domain
P_i_: inorganic phosphate
PK: pyruvate kinase
PPAR: peroxisome proliferator-activated receptor
qRT-PCR: quantitative RT-PCR
RV: right ventricular
RXR: retinoid X receptor
SV: stroke volume
TAG: triacylglycerol
UCP: uncoupling protein

## References

[1] Neubauer S. (2007) The failing heart—an engine out of fuel. N. Engl. J. Med. 356, 1140–1151 17360992 10.1056/NEJMra063052

[2] Boehm E. A., Jones B. E., Radda G. K., Veech R. L., Clarke K. (2001) Increased uncoupling proteins and decreased efficiency in palmitate-perfused hyperthyroid rat heart. Am. J. Physiol. Heart Circ. Physiol. 280, H977–H983 11179038 10.1152/ajpheart.2001.280.3.H977

[3] Cole M. A., Murray A. J., Cochlin L. E., Heather L. C., McAleese S., Knight N. S., Sutton E., Jamil A. A., Parassol N., Clarke K. (2011) A high fat diet increases mitochondrial fatty acid oxidation and uncoupling to decrease efficiency in rat heart. Basic Res. Cardiol. 106, 447–457 21318295 10.1007/s00395-011-0156-1 PMC3071466

[4] Clarke K., O’Connor A. J., Willis R. J. (1987) Temporal relation between energy metabolism and myocardial function during ischemia and reperfusion. Am. J. Physiol. 253, H412–H421 3618814 10.1152/ajpheart.1987.253.2.H412

[5] Essop M. F. (2007) Cardiac metabolic adaptations in response to chronic hypoxia. J. Physiol. 584, 715–726 17761770 10.1113/jphysiol.2007.143511 PMC2276994

[6] Hurford W. E., Crosby G., Strauss H. W., Jones R., Lowenstein E. (1990) Ventricular performance and glucose uptake in rats during chronic hypobaric hypoxia. J. Nucl. Med. 31, 1344–1351 2384802

[7] Holden J. E., Stone C. K., Clark C. M., Brown W. D., Nickles R. J., Stanley C., Hochachka P. W. (1995) Enhanced cardiac metabolism of plasma glucose in high-altitude natives: adaptation against chronic hypoxia. J. Appl. Physiol. 79, 222–228 7559223 10.1152/jappl.1995.79.1.222

[8] Ngumbela K. C., Sack M. N., Essop M. F. (2003) Counter-regulatory effects of incremental hypoxia on the transcription of a cardiac fatty acid oxidation enzyme-encoding gene. Mol. Cell. Biochem. 250, 151–158 12962153 10.1023/a:1024921329885

[9] Razeghi P., Young M. E., Abbasi S., Taegtmeyer H. (2001) Hypoxia in vivo decreases peroxisome proliferator-activated receptor alpha-regulated gene expression in rat heart. Biochem. Biophys. Res. Commun. 287, 5–10 11549245 10.1006/bbrc.2001.5541

[10] Kennedy S. L., Stanley W. C., Panchal A. R., Mazzeo R. S. (2001) Alterations in enzymes involved in fat metabolism after acute and chronic altitude exposure. J. Appl. Physiol. 90, 17–22 11133888 10.1152/jappl.2001.90.1.17

[11] Hochachka P. W., Clark C. M., Holden J. E., Stanley C., Ugurbil K., Menon R. S. (1996) ^31^P magnetic resonance spectroscopy of the Sherpa heart: a phosphocreatine/adenosine triphosphate signature of metabolic defense against hypobaric hypoxia. Proc. Natl. Acad. Sci. USA 93, 1215–1220 8577743 10.1073/pnas.93.3.1215 PMC40059

[12] Caudwell Xtreme Everest Research Group (2011) Cardiac response to hypobaric hypoxia: persistent changes in cardiac mass, function, and energy metabolism after a trek to Mt. Everest Base Camp. FASEB J. 25, 792–796 20978235 10.1096/fj.10-172999

[13] Burkart E. M., Sambandam N., Han X., Gross R. W., Courtois M., Gierasch C. M., Shoghi K., Welch M. J., Kelly D. P. (2007) Nuclear receptors PPARbeta/delta and PPARalpha direct distinct metabolic regulatory programs in the mouse heart. J. Clin. Invest. 117, 3930–3939 18037994 10.1172/JCI32578 PMC2082147

[14] Gulick T., Cresci S., Caira T., Moore D. D., Kelly D. P. (1994) The peroxisome proliferator-activated receptor regulates mitochondrial fatty acid oxidative enzyme gene expression. Proc. Natl. Acad. Sci. USA 91, 11012–11016 7971999 10.1073/pnas.91.23.11012 PMC45156

[15] Finck B. N., Lehman J. J., Leone T. C., Welch M. J., Bennett M. J., Kovacs A., Han X., Gross R. W., Kozak R., Lopaschuk G. D., Kelly D. P. (2002) The cardiac phenotype induced by PPARalpha overexpression mimics that caused by diabetes mellitus. J. Clin. Invest. 109, 121–130 11781357 10.1172/JCI14080 PMC150824

[16] Lee S. H., Wolf P. L., Escudero R., Deutsch R., Jamieson S. W., Thistlethwaite P. A. (2000) Early expression of angiogenesis factors in acute myocardial ischemia and infarction. N. Engl. J. Med. 342, 626–633 10699162 10.1056/NEJM200003023420904

[17] Willam C., Maxwell P. H., Nichols L., Lygate C., Tian Y. M., Bernhardt W., Wiesener M., Ratcliffe P. J., Eckardt K. U., Pugh C. W. (2006) HIF prolyl hydroxylases in the rat; organ distribution and changes in expression following hypoxia and coronary artery ligation. J. Mol. Cell. Cardiol. 41, 68–77 16765982 10.1016/j.yjmcc.2006.04.009

[18] Kaelin W. G. Jr, Ratcliffe P. J. (2008) Oxygen sensing by metazoans: the central role of the HIF hydroxylase pathway. Mol. Cell 30, 393–402 18498744 10.1016/j.molcel.2008.04.009

[19] Simonson T. S., Yang Y., Huff C. D., Yun H., Qin G., Witherspoon D. J., Bai Z., Lorenzo F. R., Xing J., Jorde L. B., Prchal J. T., Ge R. (2010) Genetic evidence for high-altitude adaptation in Tibet. Science 329, 72–75 20466884 10.1126/science.1189406

[20] Ge R. L., Simonson T. S., Cooksey R. C., Tanna U., Qin G., Huff C. D., Witherspoon D. J., Xing J., Zhengzhong B., Prchal J. T., Jorde L. B., McClain D. A. (2012) Metabolic insight into mechanisms of high-altitude adaptation in Tibetans. Mol. Genet. Metab. 106, 244–247 22503288 10.1016/j.ymgme.2012.03.003 PMC3437309

[21] Wu R., Chang H.-C., Khechaduri A., Chawla K., Tran M., Chai X., Wagg C., Ghanefar M., Jiang X., Bayeva M., Gonzalez F., Lopaschuk G., Ardehali H. (2014) Cardiac-specific ablation of ARNT leads to lipotoxicity and cardiomyopathy. J. Clin. Invest. 124, 4795–4806 25329697 10.1172/JCI76737 PMC4347233

[22] Krishnan J., Suter M., Windak R., Krebs T., Felley A., Montessuit C., Tokarska-Schlattner M., Aasum E., Bogdanova A., Perriard E., Perriard J. C., Larsen T., Pedrazzini T., Krek W. (2009) Activation of a HIF1α-PPARgamma axis underlies the integration of glycolytic and lipid anabolic pathways in pathologic cardiac hypertrophy. Cell Metab. 9, 512–524 19490906 10.1016/j.cmet.2009.05.005

[23] Matsushima S., Kuroda J., Ago T., Zhai P., Ikeda Y., Oka S., Fong G. H., Tian R., Sadoshima J. (2013) Broad suppression of NADPH oxidase activity exacerbates ischemia/reperfusion injury through inadvertent downregulation of hypoxia-inducible factor-1α and upregulation of peroxisome proliferator-activated receptor-α. Circ. Res. 112, 1135–1149 23476056 10.1161/CIRCRESAHA.111.300171 PMC3871171

[24] Belanger A. J., Luo Z., Vincent K. A., Akita G. Y., Cheng S. H., Gregory R. J., Jiang C. (2007) Hypoxia-inducible factor 1 mediates hypoxia-induced cardiomyocyte lipid accumulation by reducing the DNA binding activity of peroxisome proliferator-activated receptor α/retinoid X receptor. Biochem. Biophys. Res. Commun. 364, 567–572 17963722 10.1016/j.bbrc.2007.10.062

[25] Aragonés J., Schneider M., Van Geyte K., Fraisl P., Dresselaers T., Mazzone M., Dirkx R., Zacchigna S., Lemieux H., Jeoung N. H., Lambrechts D., Bishop T., Lafuste P., Diez-Juan A., Harten S. K., Van Noten P., De Bock K., Willam C., Tjwa M., Grosfeld A., Navet R., Moons L., Vandendriessche T., Deroose C., Wijeyekoon B., Nuyts J., Jordan B., Silasi-Mansat R., Lupu F., Dewerchin M., Pugh C., Salmon P., Mortelmans L., Gallez B., Gorus F., Buyse J., Sluse F., Harris R. A., Gnaiger E., Hespel P., Van Hecke P., Schuit F., Van Veldhoven P., Ratcliffe P., Baes M., Maxwell P., Carmeliet P. (2008) Deficiency or inhibition of oxygen sensor Phd1 induces hypoxia tolerance by reprogramming basal metabolism. Nat. Genet. 40, 170–180 18176562 10.1038/ng.2007.62

[26] Lei L., Mason S., Liu D., Huang Y., Marks C., Hickey R., Jovin I. S., Pypaert M., Johnson R. S., Giordano F. J. (2008) Hypoxia-inducible factor-dependent degeneration, failure, and malignant transformation of the heart in the absence of the von Hippel-Lindau protein. Mol. Cell. Biol. 28, 3790–3803 18285456 10.1128/MCB.01580-07 PMC2423296

[27] Clark R., Männikkö R., Stuckey D. J., Iberl M., Clarke K., Ashcroft F. M. (2012) Mice expressing a human K(ATP) channel mutation have altered channel ATP sensitivity but no cardiac abnormalities. Diabetologia 55, 1195–1204 22252471 10.1007/s00125-011-2428-6 PMC3296019

[28] Heather L. C., Cole M. A., Lygate C. A., Evans R. D., Stuckey D. J., Murray A. J., Neubauer S., Clarke K. (2006) Fatty acid transporter levels and palmitate oxidation rate correlate with ejection fraction in the infarcted rat heart. Cardiovasc. Res. 72, 430–437 17034771 10.1016/j.cardiores.2006.08.020

[29] Chen R. L., Ogunshola O. O., Yeoh K. K., Jani A., Papadakis M., Nagel S., Schofield C. J., Buchan A. M. (2014) HIF prolyl hydroxylase inhibition prior to transient focal cerebral ischaemia is neuroprotective in mice. J. Neurochem. 131, 177–189 24974727 10.1111/jnc.12804

[30] Thalhammer A., Aik W. S., Bagg E. A. L., Schofield C. J. (2012) The potential of 2-oxoglutarate oxygenases acting on nucleic acids as therapeutic targets. Drug Discov. Today Ther. Strat. 9, e91–e100

[31] Stubbs C. J., Loenarz C., Mecinović J., Yeoh K. K., Hindley N., Liénard B. M., Sobott F., Schofield C. J., Flashman E. (2009) Application of a proteolysis/mass spectrometry method for investigating the effects of inhibitors on hydroxylase structure. J. Med. Chem. 52, 2799–2805 19364117 10.1021/jm900285r

[32] Aksentijević D., Lygate C. A., Makinen K., Zervou S., Sebag-Montefiore L., Medway D., Barnes H., Schneider J. E., Neubauer S. (2010) High-energy phosphotransfer in the failing mouse heart: role of adenylate kinase and glycolytic enzymes. Eur. J. Heart Fail. 12, 1282–1289 20940173 10.1093/eurjhf/hfq174 PMC2990411

[33] Tian Y. M., Yeoh K. K., Lee M. K., Eriksson T., Kessler B. M., Kramer H. B., Edelmann M. J., Willam C., Pugh C. W., Schofield C. J., Ratcliffe P. J. (2011) Differential sensitivity of hypoxia inducible factor hydroxylation sites to hypoxia and hydroxylase inhibitors. J. Biol. Chem. 286, 13041–13051 21335549 10.1074/jbc.M110.211110 PMC3075650

[34] Roberts A. C., Butterfield G. E., Cymerman A., Reeves J. T., Wolfel E. E., Brooks G. A. (1996) Acclimatization to 4,300-m altitude decreases reliance on fat as a substrate. J. Appl. Physiol. 81, 1762–1771 8904597 10.1152/jappl.1996.81.4.1762

[35] Murray A. J., Cole M. A., Lygate C. A., Carr C. A., Stuckey D. J., Little S. E., Neubauer S., Clarke K. (2008) Increased mitochondrial uncoupling proteins, respiratory uncoupling and decreased efficiency in the chronically infarcted rat heart. J. Mol. Cell. Cardiol. 44, 694–700 18328500 10.1016/j.yjmcc.2008.01.008

[36] Murray A. J., Lygate C. A., Cole M. A., Carr C. A., Radda G. K., Neubauer S., Clarke K. (2006) Insulin resistance, abnormal energy metabolism and increased ischemic damage in the chronically infarcted rat heart. Cardiovasc. Res. 71, 149–157 16616054 10.1016/j.cardiores.2006.02.031

[37] Young M. E., Laws F. A., Goodwin G. W., Taegtmeyer H. (2001) Reactivation of peroxisome proliferator-activated receptor α is associated with contractile dysfunction in hypertrophied rat heart. J. Biol. Chem. 276, 44390–44395 11574533 10.1074/jbc.M103826200

[38] Caudwell Xtreme Everest Research Group (2012) Acclimatization of skeletal muscle mitochondria to high-altitude hypoxia during an ascent of Everest. FASEB J. 26, 1431–1441 22186874 10.1096/fj.11-197772

[39] Huss J. M., Levy F. H., Kelly D. P. (2001) Hypoxia inhibits the peroxisome proliferator-activated receptor alpha/retinoid X receptor gene regulatory pathway in cardiac myocytes: a mechanism for O2-dependent modulation of mitochondrial fatty acid oxidation. J. Biol. Chem. 276, 27605–27612 11371554 10.1074/jbc.M100277200

[40] Jaakkola P., Mole D. R., Tian Y. M., Wilson M. I., Gielbert J., Gaskell S. J., von Kriegsheim A., Hebestreit H. F., Mukherji M., Schofield C. J., Maxwell P. H., Pugh C. W., Ratcliffe P. J. (2001) Targeting of HIF-alpha to the von Hippel-Lindau ubiquitylation complex by O2-regulated prolyl hydroxylation. Science 292, 468-472 11292861 10.1126/science.1059796

[41] McCarthy J., Lochner A., Opie L. H., Sack M. N., Essop M. F. (2011) PKCε promotes cardiac mitochondrial and metabolic adaptation to chronic hypobaric hypoxia by GSK3β inhibition. J. Cell. Physiol. 226, 2457–2468 21660969 10.1002/jcp.22592 PMC3411281

[42] Sidell R. J., Cole M. A., Draper N. J., Desrois M., Buckingham R. E., Clarke K. (2002) Thiazolidinedione treatment normalizes insulin resistance and ischemic injury in the zucker fatty rat heart. Diabetes 51, 1110–1117 11916933 10.2337/diabetes.51.4.1110

[43] Hoppeler H., Vogt M. (2001) Muscle tissue adaptations to hypoxia. J. Exp. Biol. 204, 3133–3139 11581327 10.1242/jeb.204.18.3133

[44] Bernardo B. C., Weeks K. L., Pretorius L., McMullen J. R. (2010) Molecular distinction between physiological and pathological cardiac hypertrophy: experimental findings and therapeutic strategies. Pharmacol. Ther. 128, 191–227 20438756 10.1016/j.pharmthera.2010.04.005

[45] Braissant O., Foufelle F., Scotto C., Dauça M., Wahli W. (1996) Differential expression of peroxisome proliferator-activated receptors (PPARs): tissue distribution of PPAR-alpha, -beta, and -gamma in the adult rat. Endocrinology 137, 354–366 8536636 10.1210/endo.137.1.8536636

[46] Kashiwaya Y., Sato K., Tsuchiya N., Thomas S., Fell D. A., Veech R. L., Passonneau J. V. (1994) Control of glucose utilization in working perfused rat heart. J. Biol. Chem. 269, 25502–25514 7929251

[47] Caudwell Xtreme Everest Research Group (2014) Effects of prolonged exposure to hypobaric hypoxia on oxidative stress, inflammation and gluco-insular regulation: the not-so-sweet price for good regulation. PLoS One 9, e94915 24733551 10.1371/journal.pone.0094915 PMC3986261

[48] Holloway C. J., Cochlin L. E., Emmanuel Y., Murray A., Codreanu I., Edwards L. M., Szmigielski C., Tyler D. J., Knight N. S., Saxby B. K., Lambert B., Thompson C., Neubauer S., Clarke K. (2011) A high-fat diet impairs cardiac high-energy phosphate metabolism and cognitive function in healthy human subjects. Am. J. Clin. Nutr. 93, 748–755 21270386 10.3945/ajcn.110.002758

[49] Panagia M., Gibbons G. F., Radda G. K., Clarke K. (2005) PPAR-α activation required for decreased glucose uptake and increased susceptibility to injury during ischemia. Am. J. Physiol. Heart Circ. Physiol. 288, H2677–H2683 15665064 10.1152/ajpheart.00200.2004

[50] Sambandam N., Morabito D., Wagg C., Finck B. N., Kelly D. P., Lopaschuk G. D. (2006) Chronic activation of PPARalpha is detrimental to cardiac recovery after ischemia. Am. J. Physiol. Heart Circ. Physiol. 290, H87–H95 16155108 10.1152/ajpheart.00285.2005

[51] Luptak I., Balschi J. A., Xing Y., Leone T. C., Kelly D. P., Tian R. (2005) Decreased contractile and metabolic reserve in peroxisome proliferator-activated receptor-alpha-null hearts can be rescued by increasing glucose transport and utilization. Circulation 112, 2339–2346 16203912 10.1161/CIRCULATIONAHA.105.534594

[52] Smeets P. J., Teunissen B. E., Willemsen P. H., van Nieuwenhoven F. A., Brouns A. E., Janssen B. J., Cleutjens J. P., Staels B., van der Vusse G. J., van Bilsen M. (2008) Cardiac hypertrophy is enhanced in PPAR alpha−/− mice in response to chronic pressure overload. Cardiovasc. Res. 78, 79–89 18187461 10.1093/cvr/cvn001

